# The Cultural Validation of Two Scales to Assess Social Stigma in Leprosy

**DOI:** 10.1371/journal.pntd.0003274

**Published:** 2014-11-06

**Authors:** Ruth M. H. Peters, Dadun, Wim H. Van Brakel, Marjolein B. M. Zweekhorst, Rita Damayanti, Joske F. G. Bunders, Irwanto

**Affiliations:** 1 Athena Institute, Faculty of Earth and Life Sciences, VU University, Amsterdam, The Netherlands; 2 Faculty of Public Health, Universitas Indonesia, Depok, Indonesia; 3 Netherlands Leprosy Relief, Amsterdam, The Netherlands; 4 Centre for Disability Studies, Faculty of Social and Political Sciences, Universitas Indonesia, Depok, Indonesia; London School of Hygiene and Tropical Medicine, United Kingdom

## Abstract

**Background:**

Stigma plays in an important role in the lives of persons affected by neglected tropical diseases, and assessment of stigma is important to document this. The aim of this study is to test the cross-cultural validity of the Community Stigma Scale (EMIC-CSS) and the Social Distance Scale (SDS) in the field of leprosy in Cirebon District, Indonesia.

**Methodology/principle findings:**

Cultural equivalence was tested by assessing the conceptual, item, semantic, operational and measurement equivalence of these instruments. A qualitative exploratory study was conducted to increase our understanding of the concept of stigma in Cirebon District. A process of translation, discussions, trainings and a pilot study followed. A sample of 259 community members was selected through convenience sampling and 67 repeated measures were obtained to assess the psychometric measurement properties. The aspects and items in the SDS and EMIC-CSS seem equally relevant and important in the target culture. The response scales were adapted to ensure that meaning is transferred accurately and no changes to the scale format (e.g. lay out, statements or questions) of both scales were made. A positive correlation was found between the EMIC-CSS and the SDS total scores (r = 0.41). Cronbach's alphas of 0.83 and 0.87 were found for the EMIC-CSS and SDS. The exploratory factor analysis indicated for both scales an adequate fit as unidimensional scale. A standard error of measurement of 2.38 was found in the EMIC-CSS and of 1.78 in the SDS. The test-retest reliability coefficient was respectively, 0.84 and 0.75. No floor or ceiling effects were found.

**Conclusions/significance:**

According to current international standards, our findings indicate that the EMIC-CSS and the SDS have adequate cultural validity to assess social stigma in leprosy in the Bahasa Indonesia-speaking population of Cirebon District. We believe the scales can be further improved, for instance, by adding, changing and rephrasing certain items. Finally, we provide suggestions for use with other neglected tropical diseases.

## Author Summary

Persons affected by neglected tropical diseases, such as, Buruli ulcer, lymphatic filariasis, onchocerciasis, leishmaniasis and leprosy, can experience stigma. One important source of stigma are members in the community. Neighbours, religious leaders, and community leaders can exclude, reject, blame or devalue a person affected by one of these diseases. It is important to be able to assess this type of stigma for the prevention and management of these diseases. Assessing stigma is not an easy task. There are several instruments available, but these were developed with different aims or tested in different settings. We can use these instruments, but we need to be sure that they assess what we want them to assess and whether the instrument produces consistent results. In this paper the authors report a study that investigated the validity of two scales that assess stigma in the community towards people affected by leprosy in Indonesia. The names of the scales are Explanatory Model Interview Catalogue Community Stigma Scale (EMIC-CSS) and Social Distance Scale (SDS). The results show the two scales to be adequately valid and reliable in the target culture. There are, however, also several improvements possible and the authors provide suggestions how to incorporate these. In addition, the authors provide recommendations for the use of these scales among people affected by other neglected tropical diseases.

## Introduction

*They stood with their eyes fixed on Bengek, who crouched on the wall ready to defend himself with his spade. Slowly they retreated, for now they could all see it: he had the great sickness – the frightful sickness whose name might never be uttered.…*

*Krkek looked round and saw a heap of stones, which had all broken away from the coral-stone wall. He stooped and picked one up and threw it at the leper. Pak, too, picked up a stone – it was rough and heavy to his hand – and threw it. All men flung themselves on the stones and hurled them at the wretch on the wall. …*

*Bengek gazed at the men for a moment as though he did not realize what they were doing. Then threw away his spade and jumping of the wall ran in great bounds to the beach. …“The gods know whom they punish,” Krkek said softly. “We will go to the Pedanda [a Hindu scholarly high priest or priestess] and be cleansed.”*

*From ‘Love and death in Bali’, by Vicki Baum (1937)*

Several important aspects of leprosy are highlighted in this short book. The most prominent ones are: the fear for the disease, beliefs around causation, degradation of the person affected by leprosy and exclusion by villagers. These aspects were germane at the time the book was written. The manifestations of today are different, but resemblances remain (see Peters et al for manifestations in Cirebon District, the study area [1]). Leprosy is often seen as the archetype of a stigmatised health condition [2], [3], but is certainly not the only disease in which stigma plays a role. Other Neglected Tropical Diseases (NTDs) are, for instance, Buruli ulcer, lymphatic filariasis, onchocerciasis, leishmaniasis and Chagas disease [4]–[6]. But stigma plays also a role in many other diseases, such as tuberculosis, HIV/AIDS and mental illness [7]–[10].

Stigma is a complex construct. A variety of definitions and frameworks have been developed to operationalize the concept of stigma, as shown by Link and Phelan [11] and Yang et al [12]. Two prominent reasons for this variation are the association of stigma with an array of attributes, circumstances, health conditions and social groups, and the multidisciplinary nature of the research on stigma [11]. Not surprisingly, scholars have highlighted the lack of conceptual adequacy of the concept of stigma [13], [14]. Manzo claims that “ ‘stigma’ has become under-defined and over-used” [15]. Link and Phelan urge researchers to be clear about what they mean by stigma [11].

The focus of this paper is on the stigma of leprosy. We believe the definition of health related stigma by Weiss et al is relevant for this paper as it provides a good impression of the breadth and depth of the concept. Health related stigma is:

a social process, experienced or anticipated, characterized by exclusion, rejection, blame, or devaluation that results from experience, perception or reasonable anticipation of an adverse social judgment about a person or group [16].

More specifically, this paper is about the social stigma, also known as the public stigma, of leprosy. Social stigma exist at a group level or meso level and describes, according to Corrigan et al, “the phenomenon of large social groups endorsing stereotypes about and acting against a stigmatized group” [17]. The model of Weiss, who extended the Hidden Distress Model of Scambler [18] clearly distinguishes between those who are stigmatized and stigmatizers and unpacks both. According to this model, stigmatizers exhibit accepted, endorsed or enacted stigma. The latter is often called ‘discrimination’. Endorsed stigma refers to justifying and supporting exclusion but refraining from being actively engaged in it, while ‘accepted’ means not endorsing, but nevertheless not speaking out against the process [16]. The notion of “power” also requires some attention in the light of social stigma, for instance, Link and Phelan note that for stigma to occur “power must be exercised” [11]. Drawing on these theories we define social stigma for this paper as: the phenomenon of social groups or individuals accepting, endorsing or enacting negative attitudes that are characterized as exclusion, rejection, blame and devaluation against a stigmatized group.

Different instruments are available [19] to assess the concept of stigma. The Stigma Assessment and Reduction of Impact (SARI) Project in Indonesia wanted to determine the effectiveness of stigma reduction interventions. This is an area in which both quantitative and qualitative evidence is still lacking [20], [21]. Two promising scales were selected to assess social stigma from a community perspective: the Explanatory Model Interview Catalogue Community Stigma Scale (EMIC-CSS) and the Social Distance Scale (SDS) [22]–[24]. To our knowledge, the SDS has never been applied in the field of leprosy and the EMIC-CSS was new in its current context. Hence, there was a need to culturally validate these scales in the context of the SARI project, which is Cirebon District, Indonesia.

The cross-cultural validation of scales is a crucial process, because as unreliable and invalid scales can lead to imprecise or biased results and hence wrong conclusions. A validation process might appear to be relatively easy and straightforward, whereas is in reality it is rather complex. First, there are different orientations towards cross-cultural research; absolutism, universalism and relativism. Herdman et al write that each orientation on research has a different assumption on the extent to which ‘culture’ has an impact on the construct being measured. In the absolutist orientation, the assumptions is that the impact of culture is minimal, the universalist orientation is open to the suggestion that the impact is significant and in the relativist approach, the impact is assumed to be so substantial that “it is impossible to use standard instruments across cultures; only local instruments may be used” [25]. Second, it is necessary to be able to show equivalence between translated versions of the same questionnaire. There has, however, been confusion among scholars around the types and definitions of equivalences or the definitions and criteria to determine what are “good” measurement properties [25]–[27]. Recent initiatives such as the COSMIN study have brought greater clarity [28]. Third, from a more practical point of view, there are often limited resources (time, money, knowledge and experience) available to execute a validation study. Hence, when scales are used in a new cultural setting a cross-cultural validation is often not done or is done incompletely [27], [29]–[31].

This paper aims to investigate the cultural validity of the SDS and EMIC-CSS in Cirebon District, Indonesia using a universalist orientation. In addition, we hope to bring a contribution to the body of knowledge regarding cross-cultural validation of instruments and assessment of social stigma in general. Before the validity of scales was tested, it was important to consider where the scales originated from and how they have developed over time. Therefore, this paper starts with a brief overview of the origin and recent applications of the scales.

### Origin of the scales

#### EMIC Community Stigma Scale

In the 90s, Weiss et al identified a need for improved links between clinical, epidemiological and social science frameworks as this was believed to benefit the study of problems posed by tropical diseases. Weiss et al [22] used Kleinman's work on explanatory models [32] as a starting point and developed a framework for data collection with five key themes based on their work in the field of leprosy in India, called the Explanatory Model Interview Catalogue (EMIC). With this framework at the basis, numerous interviews have been developed. Several focus on the concept of stigma, because its association with distress and influence on illness behaviour and help seeking [22]. The interviews derived from the EMIC frequently include quantitative variables and qualitative prose [22]. Some examples in which the EMIC was used to investigate stigma clearly show its cross-cultural and cross-condition applicability: onchocerciasis in Nigeria [33], Buruli ulcer in Ghana [5], depression in India [34], leprosy, mental health and HIV in India [7], [35], [36].

To our knowledge, only two studies have developed and partially validated a stigma scale based on the EMIC specifically for assessing the perception of the community. Rensen et al [37] tested an EMIC scale with 13 items for non-affected persons (n = 165) in the field of leprosy in India. The items used a Likert response scale from 0–3. They found a sum score that ranged from 3 to 39, a median score of 21, a mean of 20 (standard deviation (SD) 9), no floor or ceiling effects and an alpha value (internal consistency) of 0.83. There was no qualitative component. Vlassoff et al [38] also developed a scale to assess stigma against the disease onchocerciasis and tested it in Nigeria, Ghana, Cameroon and Uganda. In this study, photographs and short descriptions were used. In a sample of 410 unaffected persons, they found a Cronbach's alpha of 0.76. The qualitative component had added value as it revealed aspects related to gender that were not found with the quantitative data. The version of the EMIC used in the study of van Brakel et al [36] incorporating two new items related to work was used in the study described in this paper.

#### Social Distance Scale

The Social Distance Scale originated in the Bogardus study in 1926, which was designed to measure the level of acceptability of various types of social relationships of Americans with members of common ethnic groups [39], [40]. Bogardus was introduced to the concept of social distance by Park who described it as: “grades and degrees of understanding and intimacy which characterize personal and social relations” [41]. The Bogardus scale [23] was originally a self-completing questionnaire that used a so-called Guttman scale (ranked binary answers) comprising a series of statements. In 1987, Link et al modified the Bogardus scale to understand the importance of labels attached to persons with former mentally illness [24]. This modified versions includes seven questions representing the following social relationships: renting a room, common place of work, neighbourhood, member of the same social circle, personal job brokering, marriage into one's family, and child care. Respondents could indicate to what extent they would, in the situation presented, accept the person described in a vignette, using a Likert scale. This modified scale is frequently used in the field of mental health and was the basis for this study.

To our knowledge, a thorough analysis of the psychometric properties of this modified scale has not been performed. A good internal consistency was found in several studies; with Cronbach's alphas of 0.92 [24], 0.90 [42], and 0.75 [43]. In addition, there are some results pointing to the construct validity of the scale. For instance, in a survey in Germany it was found that the perception of dangerousness and negative emotional reactions (fear, anger) were associated with a desire for increased social distance, while pro-social reactions where associated with a desire for less social distance [42]. These results were replicated (except for anger) in surveys conducted in Russia and Mongolia [44]. Similar associations with emotional reactions have also been observed in a recent survey in two large German cities [45]. There are also some results suggesting good sensitivity for change [46].

### Cross-cultural validation

The framework for cross-cultural equivalence testing used in this study, draws entirely on the work of Herdman et al [25], [30], Terwee et al [26] and Stevelink & van Brakel [29]. Five equivalences and the universalist approach are important for this study. Herdman et al note that a universalist approach:

does not make the prior assumptions that constructs will be the same across cultures and, consequently, implies a need to establish whether the concept exists and in interpreted similarly. [30]

‘Conceptual equivalence’ looks at how the concept of social stigma is conceptualized, which domains are important and at the significance accorded to these domains. ‘Item equivalence’ similarly explores how domains are conceptualized and whether items are equally relevant and acceptable in the original and the new culture. ‘Semantic equivalence’ deals with language and how meaning is transferred, for instance, whether the level of language is appropriate. ‘Operational equivalence’ concerned the suitability of the questionnaire format, instructions and mode of administration. Finally, ‘measurement equivalence’ refers to the psychometric properties (internal consistency, construct validity, agreement, reliability, floor and ceiling effects and interpretability) of the scale (for a more detailed description of each equivalence type we refer to Herdman et al [30], [35]). Table 1 describes when each of these equivalences is attained.

**Table 1:** Five categories of cultural equivalence and criteria [25], [26], [29], [30].

Equivalence	When attained?
Conceptual	Achieved when the scale has the same relationship to the underlying concept in both cultures, primarily in terms of domains included and the emphasis placed on the different domains.
Item	Item equivalence exists when items estimate the same parameters on the latent trait being measured and when they are equally relevant and acceptable in both cultures.
Semantic	Attained when meaning is transferred across languages, achieving a similar effect on respondents who speak different languages.
Operational	Realized when similar formats, instructions, mode of administration and measurement methods do not affect the results.
Measurement	Reached when the psychometric properties of the adapted version are acceptable.

## Methods

### Study site

The study area of the SARI project is Cirebon District, located on the North Coast of West Java near the provincial border with Central Java. Cirebon District has a multi-cultural population of about 2.3 million. Different languages are spoken, such as, Bahasa Indonesia (the national language), Sundanese, Javanese and Cirebonese. Annually, about 300 new leprosy cases are detected in the district and, according to key informants, there was a high level of leprosy-related stigma and limited activities to reduce this. The stigma-reduction interventions of the SARI project are implemented in 30 *kecamatan* (sub-districts).

### Research team

The SARI project team is interdisciplinary, including staff from public health, medicine, disability studies, psychology and development studies from universities in the global North and South. This validation study was executed by one postdoc researcher, three PhD students and ten research assistants from Cirebon or neighbouring districts who spoke the local languages. Four of the research assistants who interviewed community members were disabled or affected by leprosy themselves.

### Study population and sampling

The study described in this paper is part of a larger validation study that included persons affected by leprosy and community members from the 30 *kecamatan* described above. The latter group is the study population for this paper. To achieve adequate power for the various statistical calculations we estimated a sample size of at least 100 community members, with at least 50 repeated assessments to assess reproducibility [26]. The selection was done as follows; first, people affected by leprosy were invited to the *puskesmas* (Health Care Centre) for an interview. At each *puskesmas*, three persons affected by leprosy were randomly selected. For each respondent a small paper with a number was created, three papers were drawn, if the respondent came from the same village a new paper was drawn. Their *Rukun Tetangga* (RT, smallest administrative level in Indonesia approximately 10–20 households) was visited by a small team of research assistants (2–3) of the SARI project. First they visited the head of the RT to introduce themselves and explained the purpose of the project. Using convenience sampling, they then selected three community members from this RT or a neighbouring RT for the interviews. Two key persons, such as, the head of the RT, a teacher, religious leader, women's leader and one general community member about the same age and sex of the person affected interviewed that morning were selected. Data was collected during three phases: i) first validation study in August 2011, ii) baseline study from September – October 2011 and the iii) second validation study in July 2012.

### Scales

The EMIC-CSS was selected based on its prior cross-cultural and cross-condition use [5], [33]–[35], [38]. The scale has 15 items and covers areas of life that are often affected by stigma, such as concealment, avoidance, perceptions of self-worthy, shame, marriage (prospects) and work. The scale has four response options; yes (2 points), possibly (1), no (0) and do not know (0). Item 15 is scored differently; yes (0 points), possibly (1), no (2) and do not know (0). There was no qualitative component used as part of the scale, as in some previous studies [22], [38].

The SDS was selected because it measures attitudes more directly than the EMIC-CSS and had been used widely in mental health research in different countries [24], [42], [43], [47]. The scale is also short, simple and easy to contextualise, because of the use of vignettes. The SDS interview started with reading out a vignette describing a male named Rahman or female named Rahmi, depending on the sex of the interviewee. The content of the two vignettes is similar. The vignettes were developed by one of the co-authors (WvB) based on vignettes used in the field of mental health used by Angermeyer et al [42], [44], [48]. The scale has 7 items representing different degrees of social distance. The items have four response options; definitely willing (0 points), probably willing (1), probably not willing (2) and definitely not willing (3).

Both scales assess aspects of the same construct ‘social stigma’, but take a different approach; the EMIC-CSS asks how leprosy is considered in the community of the interviewee, while the SDS assesses the personal perception of the interviewee. The sum score of the individual items that all have the same weight is used as the overall score and higher scores reflect greater levels of social stigma.

### Administration

The scales are interviewer administrated. Each respondent was first asked to provide demographic information, such as, age, sex, profession and income, next the EMIC-CSS was administrated followed by SDS with vignette. This order was chosen because this sequence allows questions to go from general community perspectives to specific and personal choices and avoids ‘contamination’ of the EMIC-CSS with the vignette. When a respondent did not speak Bahasa Indonesia with sufficient fluency, the questions were translated on the spot into the first language of the respondent often Sundanese or Javanese.

### Cultural equivalence testing

To determine the conceptual, item, semantic and operational equivalence different steps were taken. First, an exploratory study took place in which 53 in-depth interviews and 20 focus group discussions (FGDs) were conducted to understand the cultural background and situation in which people lived (see for more details on the methods [1]). Second, the versions of the EMIC-CSS and the SDS that were selected for this study were translated from English to Bahasa Indonesia by someone knowledgeable regarding stigma and later back–translated to English by someone not involved in stigma research. Third, a discussion on the content of the instruments, the vignettes, the phrasing of items, and the response scales took place within the team and with experts knowledgeable on Bahasa Indonesia, leprosy, stigma and quantitative instruments. Fourth, two half-day pre-test sessions were organised with 20 participants (people affected by leprosy and with a disability). The questions of the instruments and the vignette were checked with the participants for coherence, understanding and terminology. Fifth, the research assistants of the SARI project received a full week of training in the use of the scales, with practice sessions in the office. Finally, a two-week pilot study was conducted in the study area, with daily meetings in the office. Once all scales were optimized and the interviewers felt confident, the data collection for testing measurement equivalence started followed by the baseline study. During the validation and baseline study, weekly meetings were held to discuss issues that had arisen during the interviews.

To determine the measurement equivalence, the data was entered using Epi Info for Windows, version 3.5.3, and analysed using Stata 12.1 and SPSS 21. Records were deleted from the raw database if the demographic information or a full scale was missing. Outliers were explored with descriptive statistics and box plots. Interviews conducted in a language other than Bahasa Indonesia were left out the analyses.

To provide an overview of the socio-demographic characteristics of the sample, basic descriptive statistics were calculated. The respondent was asked for either income per day or income per month; the latter was converted into one variable ‘household income per day’ by dividing the income per month by 30.5. A mean and SD were used to describe each item of the scales.

Psychometric properties were tested using appropriate statistical methods based on predefined quality criteria.

- *Construct validity:* The predefined hypothesis to assess the construct validity was that a moderate positive correlation (Spearman correlation coefficient between 0.4 and 0.8) would exist between the EMIC-CSS total score and the SDS total score. Construct validity is normally rated sufficient if at least 75% of the results are in correspondence with the hypothesis [26]. In our study only one hypothesis was formulated.
- *Internal consistency:* An exploratory factor analyses was performed to examine the dimensionality of the item set in measuring the underlying construct ‘social stigma’. An oblique promax rotation method was applied, because we expected correlations between the factors. Internal consistency was investigated by calculating Cronbach's alpha. A Cronbach's alpha between ≥0.70 and ≤0.95 was classified as good [26].
- *Reproducibility:* Community members were revisited after an interval of 3 to 29 days. This period was considered long enough to ensure that respondent would not remember their answers to the items and short enough for the stigma situation of respondents not to have changed between the assessments.
  - *°Agreement:* Agreement is tested by calculating: i) the Standard Error of Measurement (SEM) using the formula SEM_agreement_ = √σ^2^_error_, ii) the limits of agreement (Bland and Altman method) using the formula μ_difference_±1.96 * SD_difference_, iii) the Smallest Detectable Change individual (SDC_individual_) by using the formula 1.96 * √2*SEM and iv) SDC_group_ by dividing the SDC_individual_ by √n [26], [49].
  - °*Reliability:* The intra-class correlation coefficients (ICC) agreement were calculated to assess the inter-interviewer reliability of the EMIC-CSS and SDS. An ICC_agreement_ of at least 0.70 was considered evidence of good reliability [26].
- *Floor and ceiling effects:* Floor and ceiling effects are considered to be present if 15% or more of the respondents have the lowest, respectively, highest possible score on the EMIC-CSS or SDS [26].
- *Interpretability:* To determine what change in score would be meaningful, the means and SD for four subgroups were calculated (sex, age groups, level of education and key person) [26]. Only baseline survey data was used for this analysis because of the greater representativeness of the sample.

### Terminology

The term ‘assessing’ stigma is used throughout this paper, instead of for example ‘measuring’, ‘evaluating’, ‘quantifying’ or ‘rating’ stigma as this reflects best the aim of the applying the scales.

### Ethical considerations

The study was approved by the relevant offices; Ethics Committee of Atma Jaya University; Sub-Directorate for Leprosy and Yaws, Ministry of Health, Public Health Office, West Java and District Health Office, Cirebon District. Written consent was obtained from individual study subjects. The study guarantees the confidentiality of the information provided by the participants. No incentives were offered to interviewees other a small token of appreciation such as a drinking mug or t-shirt. The study abided by the CIOMS Guidelines for Research on Human Subjects [50].

## Results

### Conceptual equivalence

Based on the opinion of experts and the responses of participants of the pre-test sessions and pilot study, the domain ‘social distance’ employed in the SDS seems equally relevant and important in the target culture. Because a Likert scale is used (instead of the original Guttman scale), the fact that the type of relationship might represent different degrees of social distance is not relevant.

The EMIC-CSS assesses different aspects of a broader phenomenon that can be described as ‘perceived stigma against persons affected by leprosy’. The aspects that can be recognized in the scale applied by van Brakel et al [36] are: i) concealment (2 items), ii) process of discrediting (3 items), iii) shame and embarrassment (1 items), iv) avoidance/taking distance/isolation (2 items), v) problems with getting married or on-going marriage (2 items), vi) problems for family or other people (3 items) and vii) problems with work (2 items). The exploratory study of the SARI project described in Peters et al [1] already indicated that the aspect ‘shame and embarrassment’ and ‘avoidance/taking distance/isolation’ are relevant in the target culture. In the interviews and FGDs we found evidence for the relevance of all the other aspects and there were no indications that led us to change the emphasis placed on the aspects. In the following quote ‘concealment’ comes to the front, and at the same time reveals shame and avoidance:

*Interviewer: Did your neighbours know that you were affected by leprosy, Ma'am?*

*Interviewee: No. Nobody knew. (…) I felt ashamed of suffering from such disease.*

*Interviewer: Why did you feel so?*

*Interviewee: Of course I felt shy because people suffering from such a disease would not have friends. (…) People around here only know that I suffer from rheumatism. The problem was I was shy. I was afraid of not having any friend. Because if people know that I had the disease, my friends in the mosque would avoid me. (Interview 19, woman, 68)*

The following three quotes support the relevance of the aspect problems with getting married and on-going marriage:

*Interviewer: Before the wedding, did your husband know about your condition?*

*Interviewee: Yes, he did. … His mother thought that a disease like leprosy is contagious. … Before we got married, [name husband] and I went for blood checking in [name area] with his mother because she was afraid.*

*Interviewer: But, she agreed finally?*

*Interviewee: Yes, she did because my husband's blood type and mine are different. (Interview 11, woman, 30)*

*Interviewee: I felt sorry for my husband. I told him to marry another woman. (Interview 40, woman, 37)*

*Facilitator: Have you ever heard or do you know someone close to you whose husband left for another woman?*

*Participant: Yes, there are such cases. There was a man who decided to find another woman who was more beautiful because his wife was suffering from leprosy. (FGD 8, mothers of affected children and young women affected)*

The following quotes confirms the relevance of the aspects problems for family or other people. The first comes from a FGD among mothers of children affected by leprosy, the second from discussion among community leaders:

*Facilitator: How did your neighbours treat you—not your children? Did people in the community avoid you as well because your child or children were suffering from leprosy?*

*Respondent 1: Yes. My neighbours avoided me as well.*

*Respondent 2: They reminded their children not to accept food from our children or from us. (FGD 8, mothers of affected children and young women affected)*

*Participant: They becomes reserved and uncommunicative, shy. He himself or she herself and the family feel ashamed by the disease. One of my neighbours is behaving that way. (FGD 2, community leaders)*

The next quote illustrates the significance of the aspect process of discrediting:

*Participant: I keep thinking about how to cure the disease, how people would see and think, and whether the community will look down on my child or not. (FGD 4, mothers of children affected)*

One relatively new aspect in the EMIC-CSS is the aspect ‘problems with work’. Several studies [7], [36] have shown that this aspect is relevant. Also the data from the interviews and FGD strongly support this as shown by several quotes each highlighting a different element or perspective of this aspect. The first three quotes are from persons affected by leprosy the latter three from community members:

*Interviewee: I only hope that I could be cured. To be able to get a job. (Interview 20, male, 21)*

*Participant: I used to have a business you know and I did sell well at first. When I had the disease, people kept a distance from me. They reminded each other not to shop at my place because they might be infected by the disease. Imagine how broken hearted I was hearing that! (FGD 8, mothers of affected children and young women affected)*

*Participant: Yes. I used to work you know, but I was fired. (…) They said that they were afraid [laughs]. (FGD 8, mothers of affected children and young women affected)*

*Participant 1: If they are totally cured, I think the community will accept them.*

*Participant 2: But I don't know if they are selling a food. …*

*Participant 2: Yes, the people will still remember that person is affected by leprosy even they are totally recovered.*

*Facilitator 2: So what should the people who affected by leprosy do?*

*Participant 2: They must take another business than a food business. (FGD 1, neighbours of people affected and general community members)*

*Participant: Yes, because it is disgusting. Disgusting because she sells the food, maybe if she sells something else like flowers or other things maybe there is no disgusting feeling. (FGD 14, religious and community leaders)*

*Participant 2: No, nobody wants to buy.*

*Participant 1: Of course they feel disgusted.*

*Participant 3: Even if they are farmers, they will still feel shy. [laughs]*

*Participant 1: Because your feet are dipped in the water and people are afraid of being infected. (FGD 2, community leaders)*

### Item equivalence

Based on the opinion of experts and the response during the pre-test sessions and the pilot study there was no indication for a need to change any of the items in the SDS or in the EMIC-CSS.

### Semantic equivalence

The target population speaks different languages, but the scales are translated in Bahasa Indonesia only, because this is the national language and is most commonly spoken by the target population. Some minor changes were made in the first version of the translation to make sure that the words in the scales fitted the day-to-day language used in the people in the rural areas of Cirebon. The response options ‘possibly’ of the EMIC-CSS and ‘probably’ in SDS were difficult to translate into Bahasa Indonesia and therefore changed into ‘maybe’ translated as ‘*mungkin*’.

### Operational equivalence

Sometimes, interviewees requested to fill the forms by themselves, which was often accepted. The interviewer would be there to answer any questions. Therefore, a mixture of interviewer-administrated and self-administered form filling was used, using the same questionnaires for both. No other changes were made to the administration, formats of scales and their scoring.

### Participant characteristics

A total of 326 observations were in the initial database. Of these, 29 (8.9%) were omitted due to missing values and 38 (11.7%) were omitted due a language used other than Bahasa Indonesia. The remaining 259 community members were included in this validation study. The observations omitted differed from the main sample. The former were less frequently male (58.8% versus 62.2%), were older (mean 46.9 versus 42.1 years), more frequently married (98.0% versus 91.1%) and had fewer years of education (6.1 versus 9.1 years).

Of the 259 observations, 72 were collected during the first validation study, 142 during the baseline and finally 46 during the second validation study. Their socio-demographic characteristics are described in Table 2. The key persons in this sample were more frequently men (75% compared to 42%), had higher age (mean 44.3 versus 39.4 years), were more frequently married (96% versus 85%) and had a higher level of education (mean 10.2 versus 7.5 years) than the respondents in the ‘general’ community sample (data not shown).

**Table 2:** Socio-demographic characteristics participants.

Variables		Community members (n = 259)
Sex	Male (%)	98 (37.8)
	Female (%)	161 (62.2)
Age; mean (SD)		42.1 (12.3)
Marital status	Not married (%)	19 (7.3)
	Married (%)	236 (91.1)
	Divorced (%)	1 (0.4)
	Widow (%)	3 (1.2)
Education	Illiterate (%)	6 (2.3)
	Can read and write (%)	5 (1.9)
	Primary school (%)	99 (38.2)
	Secondary school (%)	119 (46.0)
	High school or university (%)	30 (11.6)
Education in years; mean (SD)		9.1 (4.3)
Profession*	Paid job (%)	95 (36.8)
	Own business or farmer (%)	85 (33.0)
	Housewife (%)	28 (10.9)
	Other (%)	50 (19.3)
Household income per day; mean (SD) (Rp)**		45,426 (44,888)
Key role in community e.g. village head, religious leader	Yes (%)	151 (60.4)
	No (%)	99 (39.6)
Know person affected by leprosy	Yes (%)	198 (78.9)
	No (%)	53 (21.1)

*n = 258.

**n = 243, 1 euro was equivalent to about IDR 12,000.

### Item characteristics

The mean total score of the items of the EMIC-CSS was 15.38 (SD 6.46) and ranged from 0 representing the minimum stigma score to 30 representing the maximum total score. These figures for the SDS are, respectively, 9.05 (SD 4.01) and 0 to 21. Table 3 and 4 provide the mean score per item.

**Table 3:** Descriptive statistics items EMIC-CSS (score 0–2) (n = 259).

Items	Mean	SD
E1 Would a person with leprosy keep others from knowing, if possible?	0.92	0.85
E2 If a member of your family had leprosy, would you think less of yourself?	0.63	0.85
E3 In your community, does leprosy cause shame or embarrassment?	1.42	0.80
E4 Would others think less of a person with leprosy?	1.00	0.81
E5 Would knowing that someone has leprosy have an adverse effect on others?	0.73	0.85
E6 Would other people in your community avoid a person affected by leprosy?	0.90	0.84
E7 Would others refuse to visit the home of a person affected by leprosy?	0.75	0.76
E8 Would people in your community think less of the family of a person with leprosy?	0.53	0.75
E9 Would leprosy cause problems for the family?	1.12	0.83
E10 Would a family have concern about disclosure if one of their members had leprosy?	1.25	0.80
E11Would leprosy be a problem for a person to get married?	1.41	0.73
E12 Would leprosy cause problems in an on-going marriage?	1.08	0.77
E13 Would having leprosy cause a problem for a relative of that person to get married?	0.86	0.77
E14 Would having leprosy cause difficulty for a person to find work?	1.53	0.69
E15 Would people buy food from a person affected by leprosy?	1.28	0.79

**Table 4:** Descriptive statistics items SDS (score 0–3) (n = 259).

Items	Mean	SD
S1 How would you feel about renting a room in your home to someone like Rahman/Rahmi?	1.22	0.76
S2 How about being a worker on the same job with someone like Rahman/Rahmi?	1.19	0.75
S3 How would you feel having someone like Rahman/Rahmi as a neighbour?	0.86	0.70
S4 How about having someone like Rahman/Rahmi as caretaker of your children for a couple of hours?	1.63	0.82
S5 How about having one of your children marry someone like Rahman/Rahmi?	1.76	0.83
S6 How would you feel about introducing Rahman/Rahmi to a young woman you are friendly with?	1.33	0.78
S7 How would you feel about recommending someone like Rahman/Rahmi for a job working for a friend of yours?	1.07	0.71

### Construct validity

We found a moderately positive correlation between the EMIC-CSS total score and the SDS total score (r = 0.41). We identified one outlier with contradicting total scores; EMIC-CSS total score of 0 and a SDS total score of 19. This respondent frequently answered ‘do not know’ at the items of the EMIC-CSS. Without this outlier, the correlation increased somewhat (r = 0.45). This correlation confirmed the a priori hypothesis.

### Internal consistency

Cronbach's alphas of 0.83 and 0.87 were found for the EMIC-CSS and SDS, respectively. Item E15 of the EMIC-CSS has a low item-test correlation (0.16) and item-rest correlation (0.04); if left out Cronbach's alpha of the EMIC-CSS increases slightly to 0.84.

The exploratory factor analysis for both scales showed an adequate fit as a one-dimensional scale, with a first factor explaining 77% of the score variability for EMIC-CSS and 94% for SDS. However, additional factor analysis of the EMIC-CSS also supports two factors as shown in Table 5. The first factor with 9 or 10 items and a second with 4 or 5 items. The two factors were strongly correlated (r = 0.63), supporting the presence of a single higher-order factor. Item E15 did not fit well in either scale and was therefore omitted. Cronbach's alphas for the subscales were sufficient and are provided in Table 6.

**Table 5:** 15 item EMIC-CSS exploratory factor analysis (2 factors) (n = 259).

Items	Factor 1	Factor 2
E1 Would a person with leprosy keep others from knowing, if possible?	0.36	
E2 If a member of your family had leprosy, would you think less of yourself?	0.76	
E3 In your community, does leprosy cause shame or embarrassment?	0.67	
E4 Would others think less of a person with leprosy?	0.60	
E5 Would knowing that someone has leprosy have an adverse effect on others?	0.50	
E6 Would other people in your community avoid a person affected by leprosy?	0.71	
E7 Would others refuse to visit the home of a person affected by leprosy?	0.52	
E8 Would people in your community think less of the family of a person with leprosy?	0.71	
E9 Would leprosy cause problems for the family?	0.36	0.36
E10 Would a family have concern about disclosure if one of their members had leprosy?	0.37	
E11Would leprosy be a problem for a person to get married?		0.81
E12 Would leprosy cause problems in an on-going marriage?		0.75
E13 Would having leprosy cause a problem for a relative of that person to get married?		0.63
E14 Would having leprosy cause difficulty for a person to find work?		0.68
E15 Would people dislike buying food from a person affected by leprosy?		

**Table 6:** Internal consistency subscales EMIC-CSS (n = 259).

Items	Cronbach's alpha
E1–E10	0.80
E1–E8, E10	0.78
E9, E11–E14	0.74
E11–E14	0.73

### Responsiveness

While exploring the data with frequencies and a box plot several outliers where identified and these were checked visually. Three observations seems to be errors and were therefore deleted from the database leaving in 67 repeated observations. Community members were revisited on average after 12 days, but at least after 3 and before 29 days.

The mean difference between interviewers is in the EMIC-SDD −0.52 (SD 3.37). This led to a SEM_agreement_ of 2.38, which represents 7.9% of the score range. The limits of agreement are −7.12 and 6.08. The SDC_individual_ is 6.60 and SDC_group_ is 0.81. In the SDS, the mean difference between interviewers is −0.06 (SD 2.54). The SEM_agreement_ 1.78, this is 8.6% of the total score range. The limits of agreement are −5.04 and 4.91. The SDC_individual_ is 4.94 and SDC_group_ is 0.60. For the EMIC-CSS and the SDS the test-retest reliability was above 0.70, the ICC_agreement_ is respectively 0.84 (Confidence Interval (CI) 0.75–0.90) and 0.75 (CI 0.62–0.84).

### Floor and ceiling effects

No floor or ceiling effects were identified for the EMIC-CSS of SDS. Only 2 (0.8%) of the respondents scored the lowest possible score of 0 and also 2 (0.8%) scored the highest possible score of 30 points on the EMIC-CSS. For the SDS, only 4 respondents (1.5%) had the lowest possible score of 0 and 1 (0.4) had the highest possible score of 21.

### Interpretability

The means and SD of the different subgroups of the baseline data (n = 142) show varied results as illustrated in Table 7. The mean total score of EMIC-CSS and SDS is higher in females, but the differences are very small. Among age groups, the EMIC-CSS steadily increases with age, but for SDS it slightly drops at first before increasing again. EMIC-CSS and SDS total scores follow a similar fluctuating pattern across education groups. Finally, EMIC-CSS and SDS total scores are lower fore key persons compared to the ‘general’ community.

**Table 7:** Means and SD for subgroups (n = 142).

Variables		EMIC-CSS total score mean (SD)	SDS total score mean (SD)
Sex	Male	15.37 (6.11)	9.31 (3.88)
	Female	16.12 (6.89)	10.29 (4.20)
Age group	20–29 (n = 24)	15.50 (7.41)	9.38 (4.38)
	30–39 (n = 39)	13.69 (6.60)	8.54 (4.05)
	40–49 (n = 40)	16.33 (4.91)	9.83 (3.44)
	50–59 (n = 18)	15.78 (7.28)	9.67 (4.03)
	60–70 (n = 21)	18.00 (5.99)	11.81 (4.01)
Education	Non formal education (n = 6)	18.00 (5.51)	13.67 (3.67)
	Primary school (n = 44)	16.93 (6.58)	10.80 (3.68)
	Secondary school (n = 73)	13.96 (6.02)	8.73 (3.92)
	High school or university (n = 19)	18.37 (6.16)	9.42 (4.03)
Key person	Yes (89)	15.21 (6.11)	8.87 (3.54)
	No (53)	16.36 (6.85)	11.02 (4.41)

A summary of the key findings for the two scales can be found in Table 8.

**Table 8:** Summary key psychometric properties EMIC-CSS and SDS.

Characteristics		EMIC-CSS	SDS
Number of respondents		259	259
Number of missing items		8.9%	8.9%
Internal consistency	Exploratory Factor Analysis	77% of variance	94% of variance
	Cronbach's alpha	Whole scale α = 0.83,	Whole scale α = 0.87
		Minus item 15 α = 0.84	
Construct validity		r = 0.41	
Agreement	SEM_agreement_	2.38	1.78
	Limits of agreement	−7.12 and 6.08	−5.04 and 4.92
	SDC_individual_	6.60	4.94
	SDC_group_	0.81	0.60
Reliability	ICC_agreement_ (CI)	0.84 (CI 0.75–0.90)	0.75 (CI 0.62–0.84)
Floor and ceiling effects		(0) = 0.8%	(0) = 1.5%
		(30) = 0.8%	(21) = 0.4%

## Discussion

The assessment of stigma in NTDs may serve at least four main purposes: i) increasing our understanding of NTDs and their social impact, ii) increasing our understanding of stigma and its determinants and dynamics, iii) assessing the severity of stigma over time and iv) assessing change over time [19]. This study aimed to investigate the cultural validity of the EMIC-CSS and SDS for leprosy and took a universalist orientation by assumption that culture can have a significant impact on the understanding of stigma. The results show that the EMIC-CSS and SDS are culturally valid in the present context, but there remains room for improvement as will be shown in this discussion.

A brief history of the two scales was provided at the beginning of this paper. The EMIC originates from a context comparable to the current study, namely leprosy in India [22]. The SDS, however, was developed in a very different context (USA) and with a different aim (social relations towards ethnic minorities) to which it is currently used for [39], [40]. Hence, special attention was paid to the cultural relevance of the concept and items.

### EMIC-CSS

The EMIC-CSS assesses the perceptions towards people affected by leprosy from a general community perspective (E4, E6, E7, E11, E12, E14, E15). It also addresses perceptions towards family members of a person affected by leprosy (E2, E8, E9, E13), towards other persons near a person affected by leprosy (E5) and the disease in general (E3). The EMIC-CSS assesses different aspects related to the social stigma of leprosy. This study showed that all aspects and items assessed in the EMIC-CSS are relevant in the target culture. The question whether all aspects together comprehend the concept of social stigma is more difficult and also a more theoretical/fundamental question. Experiences, such as, mocking and gossiping are real and very important experiences of people affected by leprosy in the target community [1]. These aspects are, for instance, not yet assessed and could be added to improve the content validity of the scale. Suggestions for items are: “Would other people in your community gossip about a person affected by leprosy?” or “Would other people in your community mock a person affected by leprosy?”

Two relatively new items related to ‘problems with work’ were shown to be highly relevant based on the qualitative and quantitative data. The items have high total means scores. ‘Would having leprosy cause difficulty for a person to find work?’ has a mean score of 1.53 (highest) and ‘Would people buy food from a person affected by leprosy?’ has a mean score of 1.28 (reverse coded; fourth highest). The psychometric property results, however, show that the last item does not fit in the scale. This item might be an early sign of stigma, it may be scored positive while items are scored negative. We have considered ‘Would people dislike buying food from a person affected by leprosy?, but the translations of this question caused some confusing in Bahasa Indonesia. An option to pilot in the future would be: ‘Would having leprosy cause difficulty for a person to sell food?’ For now, we recommend researchers who wish to validate or apply the EMIC-CSS in their study to include this item if it is considered culturally relevant. Investigators who are particularly interested in this item may want to develop and test a set of questions that assess the same activity in different ways.

In our opinion, question 5 ‘Would knowing that someone has leprosy have an adverse effect on others?’ is a rather abstract question in particular the ‘others’ part could have multiple interpretations. Although this did not cause challenges in our study, we would recommend caution when piloting this question to make sure the question is phrased in clearly understandable terms.

The construct validity of the EMIC-CSS (and automatically also of the SDS) was supported by the moderately positive correlation between the EMIC-CSS total score and the SDS total score. Cronbach's alpha found in this study (0.83) is comparable to the values found in other studies [37], [38]. The exploratory factor analysis indicates an adequate fit for a one-dimensional scale, with a first factor explaining 77% of the score variability. We conclude, therefore, that the internal consistency is good. The factor analysis and internal consistency analysis illustrate that two shorter versions of the EMIC-CSS may also be of value; one with 9–10 items that could be conceptualised to assess ‘perceived attitudes towards persons affected by leprosy’ and another with 4–5 items that would assess ‘perceived behaviour towards persons affected by leprosy’. The responsiveness of the EMIC-CSS was sufficient in this study. For evaluation purposes a small measurement error is required, as one wants to be able to distinguish clinically important change from measurement error [51]. The SDC of the EMIC-CSS was small at the group level (0.81 out of a score range of 30), but larger at the individual level, which means that at individual level, large score differences are required to demonstrate changes, while at group level, small score differences will already be sufficient. With an ICC_agreement_ of 0.84 the reliability is good. The absence of floor and ceiling effects was akin to other studies [37]. For interpretability, the SDC should ideally be compared with the score difference representing Minimally Important Change (MIC). However, this figure is not yet available for the measures under study. We agree with de Vet et al (22) and prefer not to calculate a MIC based on statistical tests but to use an anchor method. Hence, we underline the importance of future research to investigate the MIC for this scale.

The EMIC-CSS as used in the SARI project did not include a qualitative component as described by Weiss [22]. We would like to underscore that we do value qualitative methods very much, especially in the context of the concept of stigma. In the SARI project we use separate methods for qualitative data collection.

This study has shown that the EMIC-CSS is adequately culturally valid in the field of leprosy in Cirebon District. The scale could easily be adapted to other NTDs. This has indeed been done already in the case of onchocerciasis and Buruli Ulcer [5], [38]. Certain items can be more or less relevant in different conditions. For instance, the item related to food might be less relevant when there are no feelings of disgust or fear for infection, as in vector-borne diseases.

### Social Distance Scale

The SDS assesses the perceptions of the interviewee towards people affected by leprosy by asking how they feel regarding different types of social relationships (e.g. neighbours, caretakers, colleagues). The concept of social distance has been studied in several countries around the globe, including Argentina [52], Japan and China [53], Nigeria [54], Germany [42], and Egypt [55]. This confirms its cross-cultural value. The cross-cultural validation of the SDS has to our knowledge not received any attention and, therefore, this is the first study of its kind. The current study has shown that the concept of social distance and the different types of relations used in the items of the scale are relevant and understood in Cirebon District.

In retrospect, the item ‘renting a room to an affected person’ is less appropriate in the context of Cirebon District. Difficulties did not emerge with this item during the pilot, validation or baseline study. However, it is not a common practice for community members in the primarily rural study area to rent a room to somebody. Respondents could envision the situation, but were by and large not acquainted with the practice themselves. To make the scale more appropriate for Cirebon District or similar contexts, a replacement item is suggested. A first exploration with the SARI research assistants resulted in the following suggestion: ‘How would you feel visiting the house of someone like Rahman/Rahmi?’ It important to note that the item renting a room seems appropriate in other contexts in Indonesia. For instance, in Yogjakarta, a university city, many households offer *kost* (rooms for rent).

The internal consistency of the SDS in this study was good. Cronbach's alpha (0.87) was equivalent to the alphas found in other studies [24], [42], [43]. Factor analysis suggested one factor, which accounted from 94% of the variance. The SDC of the SDS was small at the group level (0.61 out of score range of 21), but larger at the individual level (4.98) resulting in the implications as described for the EMIC-CSS. The ICC_agreement_ was 0.75, indicating good inter-interviewer agreement. For interpretability a MIC is needed, which is unfortunately not yet available.

This study demonstrated that also the SDS is adequately culturally valid in the context of leprosy in Cirebon District. The scale can be easily adapted to other NTDs, by changing the vignette. The items need to be checked for relevance and appropriateness in the new target culture.

### Limitations

First, the convenience sampling used, the difference between observations omitted and the main sample and the high proportion of key persons are weaknesses in this study. Ameliorating circumstances are the size and diversity of the sample and the fact that the key persons are likely to know and represent well the views of their community regarding leprosy. The influence of the sampling bias on the results presented in this paper are in our point of view minor. We do not expect any influence on the conceptual, item, semantic and operational equivalences, due to the fact that the exploratory study included ‘general’ community members, the pre-test sessions were with people affected by leprosy and disabled people and the still relatively large group (99) of ‘general’ community members in the sample. We expect an influence on the measurement equivalence, but only on the interpretability as illustrated in the results; a higher total score of the EMIC–CSS and SDS for the ‘general’ community compared to key persons. Second, the effect of the mixture of interviewer-administrated and self-administrated is difficult to ascertain, because not all research assistants did this and those who did, did not do it with all respondents. Given that the same questionnaires were used and that the interviewer was present while the respondent filled in the questionnaire, we do not expect an important influence. Third, the fact that we were able to only validate Bahasa Indonesia versions of the EMIC-CSS and SDS is another weakness. A substantial group of people in Cirebon District do not speak Bahasa Indonesia sufficiently and to assess the level of social stigma in these groups, scales in different languages will still be needed.

### Reflections on the validation process

Some reflections on the process follow. First, as mentioned in the introduction, the validation of scales is a crucial process as unreliable and invalid scales can lead to wrong conclusions. This places a responsibility on researchers who intend to use an instrument in a new cultural setting or with a different target group to test the validity of the instrument using state-of-the-art qualitative and quantitative methods. Tools like the Herdman-Stevelink framework are helpful in conducting such a validation study [29], [30].

Second, a valid scale does not mean a perfect scale that we should set in stone and leave untouched. This paper shows that although the scales are valid, there remain several points for improvements. Several of these suggestions came from observations and reflections after the testing, training and piloting phases. We would like to recommend other researchers also to continue reflecting on valid scales as this might generate valuable insights and lessons in the future.

Third, can the construct of stigma be assessed and, if so, how can this best be done? Opinions differ within our own team and these can be linked to our different scientific disciplines and epistemologies. However, we all agree that a combination of qualitative and quantitative methods offer the richest perspective. In relation to stigma-reduction interventions, the data that comes from quantitative assessments have particular value in determining the effectiveness of such interventions in groups and when generalizability is important. Qualitative assessment is particularly valuable when we look at individuals and want to understand the changes (and underlying reasons for these) interventions brought in their lives.

Fourth, we consider it important to reflect on the impact assessing stigma has on the interviewee. It goes without saying that an instrument or the way data is collected should not create concern or discomfort regarding people affected by leprosy. This is why, in this type of research, the attitudes, understanding and skills of the interviewers and hence their training is crucial. Also the code of conduct of the team, for instance, on how to deal with questions about leprosy from the interviewee is vital. Phrasing questions in scales more positively, e.g., ‘Do people in your community support people affected by leprosy?’ Or assessing different constructs, such as social closeness, inclusion and care would be interesting topics for future research.

Fifth, Parker and Aggleton noted that the way one conceptualizes and investigates the construct of stigma influences forms of intervening [13]. To assess social stigma, the concept of stigma needs to be conceptualized, but this simplification of a complex construct should then not dictate other activities in the field of stigma. Simplification for the purpose of quantitatively assessing the effect of interventions is valid. However, when designing or implementing stigma reduction interventions, we believe that we need to step back, appreciate and take into account the complexity of the concept of stigma.

### Conclusion

According to current international standards, our findings indicate that the EMIC-CSS and the SDS have adequate validity to assess social stigma of leprosy in the Bahasa Indonesia-speaking population in Cirebon District. However, these findings cannot be generalized to other NTDs, countries or even other provinces in Indonesia that are culturally different, such as Papua, Sulawesi, and Nusa Tenggara, where they would need to be re-validated. We believe the scales can be further improved and we have provided several suggestions in the discussion. With some adaptations the scales can be validated for other NTDs.

## Supporting Information

Checklist S1 STROBE checklist.
(DOCX)
